# Complete genome sequence of *Bacillus subtilis* GL-4

**DOI:** 10.1128/mra.00550-24

**Published:** 2025-01-15

**Authors:** Shaomin Qin, Wenting Zeng, Jue Wei, Fenglian Chen, Ling Ma, Shuying Qin, Jun Lin, Lishi Xu, Peng Zhu, Jinfeng Liu

**Affiliations:** 1 Guangxi Key Laboratory of Veterinary Biotechnology, Key Laboratory of China (Guangxi)-ASEAN Cross-border Animal Disease Prevention and Control, Ministry of Agriculture and Rural Affairs of China, Guangxi Veterinary Research Institute, Nanning, China; 2 Anzhou District Agriculture and Rural Bureau, Mianyang, China; 3 Guangxi Key Laboratory of Beibu Gulf Marine Biodiversity Conservation, Beibu Gulf University, Qinzhou, China; Indiana University Bloomington, Bloomington, Indiana, USA

**Keywords:** applied microbiology

## Abstract

A cellulase-producing *Bacillus* was previously isolated from the intestinal tract of domestic bamboo rat and identified as *Bacillus subtilis* GL-4. In this study, we present the complete genome sequence of *B. subtilis* GL-4. The genome is 4,271,214 bp long, with a guanine-cytosine content of 43.45%.

## ANNOUNCEMENT

Previously, cellulase-producing *Bacillus subtilis* GL-4, isolated on de Man-Rogosa-Sharpe (MRS) medium (Beijing Luqiao Microbial Technology, Beijing, China) at 37°C for 24 h from the intestinal tract of domestic bamboo rat, was preliminarily screened by Congo red plate and identified as *B. subtilis* via physiological and biochemical tests and 16S rDNA PCR method (1). The endoglucanase and filter paper enzyme activities of this strain were 49.32 and 25.13 U/mL, respectively (1). In our previous study, we reported that *B. subtilis* GL-4 exhibits good tolerance to low pH (pH 2.5 and 3.5) and 0.50% bile salts and that it is a potential probiotic candidate strain (2). In the present study, we revealed the complete genome sequence of *B. subtilis* GL-4. For this, the *B. subtilis* GL-4 strain was cultured in MRS medium at 37°C for 24 h. TaKaRa MiniBEST Bacteria Genomic DNA Extraction Kit (v.3.0) was used according to the manufacturer’s instructions to extract the total genomic DNA of *B. subtilis* GL-4 from the collected bacterial culture fluid.

The PacBio RS II and Illumina Hiseq 4000 sequencing platforms were utilized to sequence the complete genome of *B. subtilis* GL-4 at Beijing Genomics Institute (Shenzhen, China). The libraries for PacBio sequencing and Illumina sequencing were prepared using the SMRTbell Express Template Prep Kit (v.2.0; PacBio, USA) and Nextera XT DNA Library Preparation Kit (Illumina, USA), respectively, according to the manufacturer’s instructions. For the PacBio platform, genomic DNA was sheared using Covaris g-TUBE; sheared DNA was purified using 0.45× AMPure PB beads; four SMRT cells with zero-mode waveguide sequencing arrays were used to generate the subread set. Through PacBio sequencing, 697,148 subreads (8,065,230,617 bases, with 1,888× coverage and a mean subread length of 11,568 bp) were obtained. In contrast, 8,764,210 reads (average insert size 350 bp, with 303× coverage) were obtained from Illumina sequencing. PacBio subreads (length <1 kb) were removed. Quality control and adapter trimming were performed using FastQC (v.0.73) and Trimmomatic (v.0.38.1) (3). Self-correction was performed using Falcon (v.0.3.0) and Proovread (v.2.12) (4). The Celera Assembler (v.8.3) (5) was used to assemble draft genomic unitigs, which are uncontested fragment groups, against a high-quality corrected circular consensus sequence subread set. To improve the accuracy of the genome sequences, single-base corrections were made using GATK (v.1.6–13) (5). To identify plasmid presence, SOAP (v.2) (6) was used to map the filtered reads to a bacterial plasmid database (http://www.ebi.ac.uk/genomes/plasmid.html). Gene prediction and genome annotation were performed using Glimmer (v.3.02) (7) and Diamond (v.3.12) (8), respectively. Default parameters were used except where otherwise noted.

The complete genome sequence of *B. subtilis* GL-4 was noted to be 4,271,214 bp long, with a total guanine-cytosine content of 43.45%. Plasmid was not detected in the genome. In total, 4,253 protein-coding sequences, 30 rRNAs, and 87 tRNAs were identified on the chromosome, which were annotated. Genome sequence analysis revealed that *B. subtilis* GL-4 comprises cellulose degradation-associated genes, including three endo-β-1,4-glucanase (EC 3.2.1.4) and five β-glucosidase (EC 3.2.1.21) genes. Furthermore, the genome comprises genes associated with the degradation of other nondigestible carbohydrates, including mannan and xylan.

## Data Availability

The complete sequence of *Bacillus subtilis* GL-4 has been submitted to GenBank and deposited under accession number CP104097. The BioProject and BioSample accession numbers are PRJNA877089 and SAMN30680087, respectively. The raw sequence reads are available under Sequence Read Archive accession numbers SRR29245815 (Illumina) and SRR29261860 (PacBio).
